# The Crystallisation, Microphase Separation and Mechanical Properties of the Mixture of Ether-Based TPU with Different Ester-Based TPUs

**DOI:** 10.3390/polym13203475

**Published:** 2021-10-10

**Authors:** Yu-Hui Que, Ying Shi, Li-Zhi Liu, Yuan-Xia Wang, Chen-Chen Wang, Hai-Chao Zhang, Xu-Yang Han

**Affiliations:** 1 Advanced Manufacturing Institute of Polymer Industry, Shenyang University of Chemical Technology, Shenyang 110142, China; queyuhui0922@hotmail.com (Y.-H.Q.); violetlj@yahoo.com (L.-Z.L.); wangyuanxia@aliyun.com (Y.-X.W.); wangchenchne@126.com (C.-C.W.); zhanghc1020@163.com (H.-C.Z.); hanxuyang0227@163.com (X.-Y.H.); 2 College of Materials Science and Engineering, Shenyang University of Chemical Technology, Shenyang 110142, China

**Keywords:** thermoplastic polyurethane, compatibility, microphase separation, mechanical properties

## Abstract

The difference in compatibility at the molecular level can lead to a change of microphase separation structure of thermoplastic polyurethanes blend systems, which will improve their thermal and mechanical properties. In this study, TDI-polyester based TPU was blended with MDI-polyether-based TPU and MDI-polyester based TPU, with different ratios. In the blend system, the obvious reduction of the melting temperature of the high-temperature TDI-polyester based TPU component indicates its hard segments can be mutually integrated with the other component. For TDI-polyester based TPU/MDI-polyether based TPU blends, their similar hard segment ratio and similar chemical structure of the soft segment give the molecular chains of the two components better compatibility. The aggregation structure of the two kinds of chains can rearrange at the molecular level which makes the hard domains mutually integrate to form a new phase separation structure with larger phase region distance. As a result, the yield strength of this blend increased by almost 143% when the elongation at break was only reduced by 12%. In contrast, the other group of blends still partly maintain their respective micro domains, forming a weak interface and leading to a decreased of elongation at break.

## 1. Introduction

TPU is a linear segmented-block copolymer composed of alternating hard (adduct of di-isocyanate and small glycols) and soft (e.g., polyester, polyether) segments connected by urethane groups (–NH–COO–). Glassy or crystalline hard segment domains act as physical crosslinks and reinforcing fillers, whereas the soft-segment phase introduces flexibility. However, the two chains are usually thermodynamically incompatible, which leads to microphase separation [1,2,3,4]. Numerous studies have reported a relationship between the mechanical properties and the microstructure aggregate structures of TPU [5,6,7,8,9,10,11,12,13,14,15,16]. In previous reports, the microphase separation structures of TPU have been controlled by adding different methylene chain units on the same soft segments of TPU [5] to compare the effects of different soft segments with the same molecular weight [6], and by regulating the hydrogen bonds between the soft and hard segments [7]. These manipulations are intended to improve the mechanical properties of TPU.

The performance of TPU can be improved not only by controlling its structure, but also by blending TPU with different materials. A large number of literature reports exist which show that blended polymers provide unusual combinations of mechanical [17], thermal, chemical [18], conductive [19] and morphological properties by compensating for the deficiencies of the individual precursors [20,21]. In blends, compatibility is an important consideration. Santra et al. [22] revealed that polyether based TPU and poly(ethylene-co-methyl acrylate) (EMA) blends are miscible throughout the entire composition range via hydrogen bond formation, thus improving oil and solvent resistance properties. Maity et al. [23] blended EVA with TPU to improve the elasticity and solvent resistance of the original TPU. The compatibility was improved by preheating technology and a heating-curing agent. Bajsio et al. [24] blended PP with TPU to reduce the fabrication costs, but TPU and PP are poorly compatible because they lack interfacial adhesion; therefore, brittle fracture behaviour occurred over a wide blend-composition range. Wang et al. [25] added maleic anhydride grafted polyethylene oxide (PEO-g-MA) as a compatibiliser to improve the compatibility (and hence the tensile strength and elongation) of the EPDM/TPU blend system.

Although TPU has been frequently blended with polyolefin [24,26], engineering plastics [27,28,29], thermoplastic elastomers [30], and rubber [31], the blending of two different TPUs has rarely been reported. Zhang et al. [32] separately blended and copolymerised polyether and polyester thermoplastic polyurethane elastomers (TPUs) with the same hard-segment structure. They reported a higher thermal decomposition temperature of the co-polymerised C-TPU than of B-TPU and C-TPU formed by copolymerising the soft segments (polyether and polyester), which they attributed to better microphase separation of the soft segments. Introducing the polyether-type soft segment destroys the regularity of the soft segments and weakens the hydrogen bonds between the polyester segments and the hard segments, thus enhancing the phase-separation structures and improving heat resistance. In a blend of two TPUs, microphase separation is caused by thermodynamic incompatibility of the soft and hard segments. Other compatibility problems occur between the soft and hard segments and the second component, which complicates the research of TPU blend systems [33]. Compatibility further depends on the rheological properties of TPU. The polar nature of the urethane segments results in a strong mutual attraction, aggregation, and ordering into crystalline and paracrystalline domains in the mobile soft segment matrix. The abundance of urethane hydrogen atoms, as well as carbonyl and ether oxygen partners, permits extensive hydrogen bonding among polymer chains, which apparently restricts the mobility of the urethane chain segments in the domains. Therefore, the microphase separation structure of TPU is expected to affect the rheological properties of the blend [34,35]. In the present study, the microphase separation structure was observed after blending different soft and hard components of TPU, and the influence of microstructural changes on the thermal and mechanical properties of TPU were analysed. This is of guiding significance for the in-depth study of the blending of different types of TPU and the control of material properties through structure.

In this paper, a TDI-type ester-based TPU was blended with an MDI-type ester-based TPU or an ether-based TPU. The microphase separation structures were controlled by comparing the effects of blending the ester-based and ether-based soft segments and MDI and TDI hard segments. When the melting point of the blend is not significantly different from those of its constituents and the elongation at break decreases slightly, the tensile strength is significantly improved.

## 2. Experiment

### 2.1. Materials and Blend Preparation

Three grades of TPU were used in the experiment (Ester-based 260 Covestro Germany, Ester-based C85A BASF Germany, Ether-based 1180A BASF Germany). Table 1 summarises the primary information of the three raw materials. The ESC85A/ES260 blends (70/30, 50/50 and 30/70 wt.% compositions) and ET1180A/ES260 blends (70/30, 50/50 and 30/70 wt.% compositions) were prepared in a TDS-20B co-rotating twin-screw extruder. Table 2 summarises the blend compositions used in this study.

### 2.2. Gel Permeation Chromatography

Molecular weight and molecular weight distribution were measured using the Agilent (Model 1260)(Santa Clara, CA, USA) equipped with Styragel-HR-5E and Styragel-HR-2 columns. The mobile phase was 0.7 mL·min^−1^ tetrahydrofuran (THF), and the column temperature was maintained at 35 °C. Polystyrene standards with varying molecular weights were used. Table 1 shows the molecular weights obtained from the tests.

### 2.3. Differential Scanning Calorimetry (DSC)

Thermal transitions in a specimen were determined using the Q100 system (TA, New Castle, DE, USA). The analysis was carried out in a nitrogen atmosphere with a 50 mL·min^−1^ flow rate and temperature ranging from −50 to 240 °C. The sample sizes used ranged from 5–8 mg; samples were kept in hermetically sealed aluminium pans.

### 2.4. Wide-Angle X-ray and Small-Angle X-ray Scattering

SAXS and WAXD measurements were performed at the Beijing Synchrotron Radiation Facility’s synchrotron beamline 1W2A in China. The energy of the X-ray radiation was 8.052 keV and had a wavelength of 0.1542 nm. For WAXD collection, Mar165-CCD (Mar USA, Norwood, NJ, USA) with a resolution of GBP 100 mm (FWHM) was set at a sample-detector distance of 89.14 mm in the direction of the beam. The exposure time was set to 20 s. The collected data were corrected for air background before any analysis.

The beam-stop size was set at 4 mm for SAXS measurements, and the distance between the sample and the detector was 1598.62 mm. The Mar165-CCD detector collected scattering patterns. The position of the peak, q_max_, is related to the long period, L, by Bragg’s law: L = 2π/q_max_, where q is the scattering vector, and is defined as q = 4π (sin θ)/λ, where λ is the X-ray wavelength and θ is half of the scattering angle (2θ) [7]. The collimation, specimen chamber and flight tube before the detector were evacuated to minimise air scattering.

### 2.5. Atomic Force Microscope (AFM)

The morphology of TPUs was observed using an SPM-9700 Origin AFM system (Shimadzu Instruments Co., Kyoto, Japan) in phase diagram mode. A 1 mm thin slice of the sample was used. Before scanning, the sample’s surface was etched in tetrahydrofuran (THF) solvent for 30 min to remove impurities on the surface. The scanning range was 2.5 μm × 2.5 μm.

### 2.6. Mechanical Testing

A CMT (Model 4000) tensile machine was used to measure mechanical properties. The tests were performed at ambient temperature (24 °C) and a 100.00 mm/min crosshead speed. For each blend, at least five test specimens of 20.00 mm × 4.0 mm × 0.75 mm were tested, and average values were calculated.

### 2.7. Hardness Test

The samples’ hardness was measured using Shore D scales. The Shore D hardness of the samples was determined at room temperature using a Shore D hardness-testing machine (Bareiss HPE Ⅲ, GER) in accordance with the ASTM D2240 standards. The hardness value was determined by the penetration of the Durometer indenter foot into the sample.

## 3. Results and Discussion

The change of TPU structure has a significant impact on its performance (thermal and mechanical). The effects can be quite different for TPUs’ different polyol type and composition. In the present paper, TPUs’ crystallization kinetics and melting behaviours were studied with DSC, and the microphase separation structure was studied with SAXS and AFM. The effect of the TPU microphase separation structure on the mechanical properties of the blend was studied with an Instron tensile tester.

### 3.1. The Non-Isothermal Crystallization Dynamics of TPU and Their Blends

**Figure 1:**
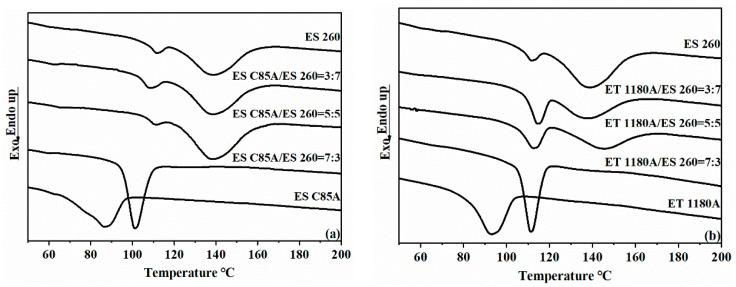
DSC curves showing the non-isothermal crystallisation of TPU and TPU blends during the cooling process: (**a**) ESC85A/ES260 blends, (**b**) ET1180A/ES260 blends. The single crystalline peak in the 30% ES260 blends is similar to that of pure TPU, indicating partial compatibility of the hard segments.

The non-isothermal crystallization of TPU and their blends were studied with DSC during a cooling process. The cooling traces of ESC85A/ES260 blends and ET1180A/ES260 blends are shown in Figure 1a,b, respectively. In Figure 1, a broad exothermic peak at 138.7 °C of ES260 is observed during cooling at a rate of 10 °C·min^−1^, which is the TDI crystallisation peak in the hard segment of ES260. It also shows a weak crystallisation peak at 111.6 °C, which corresponds to the crystallisation peak of the short TDI segment in ES260. However, the blend with 70 wt.% ESC85A (70ESC85A/30ES260) shows a significant, single crystallization peak at 101.2 °C during cooling (Figure 1a), close to the crystallisation temperature of the short TDI segment domain. The changed crystallization peak and temperature of TPU in this blend can be attributed to the partial compatibility between the hard segments. In fact, the hard segment structure of TPU has changed after blending, making the original ES260 crystallization peak disappear and significantly increasing the crystallization temperature of ESC85A. These results suggest that there is a coupling effect between the hard segments at the molecular level, allowing ESC85A to crystallize together with the short TDI domains within a temperature range. The ET1180A/ES260 blends (Figure 1b) also showed a similar trend.

### 3.2. The Melting Behavior of TPU and Their Blends

**Figure 2:**
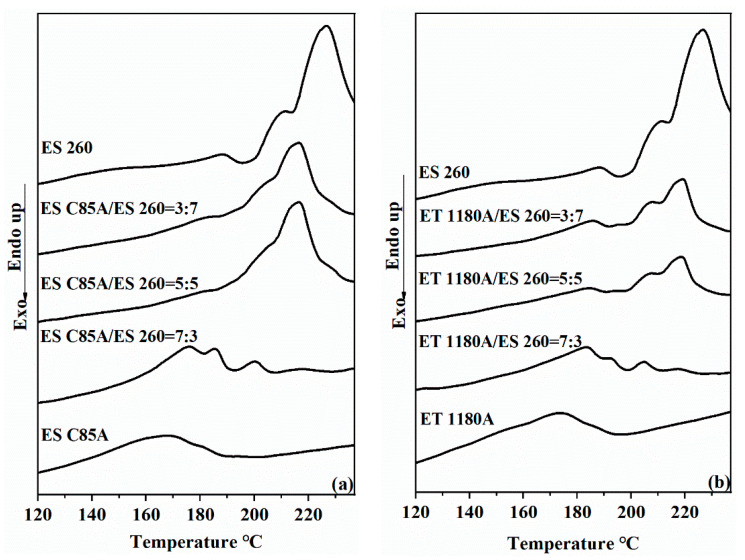
The melting behavior of TPU and their blends were studied with DSC during a heating process at a rate of 10 °C·min^−1^: (**a**) ESC85A/ES260 blends, (**b**) ET1180A/ES260 blends. The blend of 30% ES260 showed multiple melting peaks at much lower temperatures, indicating that the two components had good compatibility. The molecules of ES260 can follow ESC85A and ET1180A to crystallize at lower temperatures.

**Figure 3:**
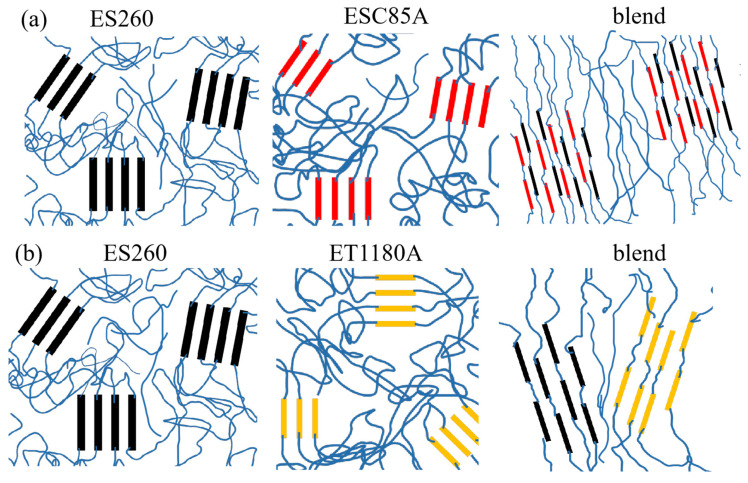
The microstructure and structure evolution of neat TPU and TPU blends. (**a**) ES260 containing TDI with a hard segment content of 33% (black), ESC85A containing MDI with a hard segment content of 33% (red). In the blend system, the aggregation structure of the two kinds of chains rearranges at the molecular level. The hard segment domain of the ESC85A/ES260 blend comprises both ESC85A and ES260 hard segments, forming new phase separation structures with larger phase region distances; (**b**) ES260 containing TDI with a hard segment content of 33% (black), ET1180A containing MDI with a hard segment content of 25% (yellow). A relatively lower compatibility of the hard domains of the ET1180A/ES260 blend, though mutually integrated to some extent, still partly maintain their respective micro domains.

The thermal properties of the ESC85A/ES260 and ET1180A/ES260 blends were derived from the DSC curves obtained with heating at 10 °C/min. The samples were initially heated to 200 °C, held for 5 min, and then cooled to −50 °C. The reheating thermograms of the ESC85A/ES260 and ET1180A/ES260 samples are shown in panels (a) and (b) of Figure 2, respectively. In Figure 2a, the large and sharp melting peak of ES260 at 228.2 °C is attributable to melting of the ES260 hard segments (TDI). Meanwhile, the relatively broad, flat peak of ESC85A at 167.1 °C corresponds to the melting of the ESC85A hard segments (MDI). When the ES260 content in the blends exceeded 50%, the secondary heating curve showed a melting peak at 216.7 °C. In terms of shape and melting temperature, this peak resembled the melting peak of ES260, indicating that at this ratio, the thermal behaviour of the blend was comparable to that of ES260. Surprisingly, multiple melting peaks were observed in the DSC curve of ESC85A/ES260 = 7:3. The melting peak of neat ES260 (at 228.2 °C) was very weak in this blend, with an area ratio well below 30%, and the melting temperature of the blend was 9 °C higher than that of neat ESC85A. This result shows that the hard segments of ES260 and ESC85A had fused and exerted a coupling effect. The TDI crystals in the ES260 component were then destroyed by entry of the MDI phase of ESC85A into the TDI phase region of ES260. This result confirms the high compatibility of ESC85A and ES260, which can also be explained by the phase structure model (Figure 3). After adding 30% ES260, the melting point of the blend increased, the chain segments grew, and the crystal particles enlarged. These behaviours manifested as an increased hard segment aggregation area in Figure 3. ET1180A/ES260 blends (Figure 2b) showed a similar overall trend.

### 3.3. Microphase Separation Structure of TPU and Their Blends

**Figure 4:**
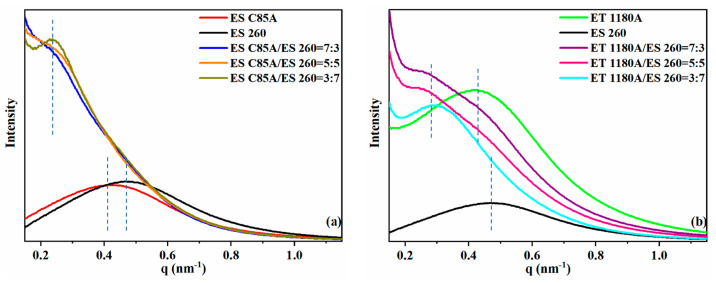
Linear SAXS profiles of neat TPU and TPU blends. (**a**) ESC85A/ES260 blends, (**b**) ET1180A/ES260 blends. The appearance of new imperfect scattering peaks of (**a**) at low angles indicates the formation of a new phase region structures. Relatively ordered scattering peaks (**b**) appear at low angles. The small change of the blends’ scattering peaks compared with the neat TPU indicates the small change of phase structure. The position of the scattering peak is denoted by the blue dotted line.

**Figure 5:**
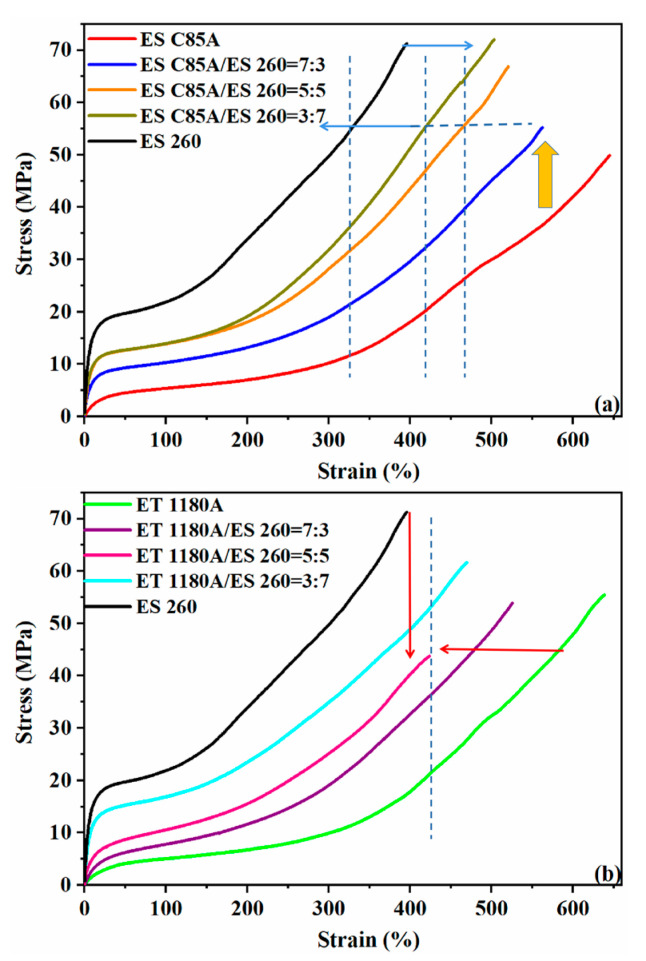
Stress vs. elongation traces of selected TPUs; change in behaviour with polyol type and composition. (**a**) ESC85A/ES260 blends. The change of the microphase separation structure has optimized the mechanical properties of the blend to varying degrees. The yield strength of the 30% ES260 blend is increased by 143% (3.5 vs. 8.5 MPa) when the elongation at break is only reduced by 12%, which has been marked with an orange arrow in the figure; (**b**) ET1180A/ES260 blends. Compared with the former, the improvement of mechanical properties is not obvious, especially in blends under a 50% ratio (marked with red arrows), the performance is significantly reduced, indicating a relatively lower compatibility.

The microphase separation structures in the neat TPU and TPU blends were also studied with in situ synchrotron SAXS. Figure 4 shows the SAXS profiles of the ESC85A/ES260 and ET1180A/ES260 blends. In Figure 5a, the near-perfect scattering peaks of ESC85A and ES260 around q = 0.42 nm^−1^ correspond to the distance between the hard-segment domains dispersed among the soft segments. After blending, the characteristic scattering peaks of ESC85A and ES260 almost disappeared, and a new imperfect scattering peak appeared at q = 0.24 nm^−1^. The peak shape of the scattered signal was poorly defined, particularly in ESC85A/ES260 = 7:3, indicating no macroscopic phase separation between ESC85A and ES260. The phase regions of the highly compatible ESC85A and ES260 recombined into a new scattering structure (see Figure 3 for the model). However, in Figure 4b, the scattering peaks of the blends shifted to a lower angle and were not a simple superposition of the peaks of neat ET1180A and ES260. Therefore, no macroscopic phase separation occurred in this blend system. The ET1180A/ES260 blend system maintained a slightly better peak shape, indicating that although the phase region of the ET1180A/ES260 blends became less ordered, the change was less dramatic than in the ESC85A/ES260 system, and the compatibility was lower than in the ESC85A/ES260 system.

### 3.4. Mechanical Properties of TPU and Their Blends

The mechanical properties of ESC85A/ES260 blends and ET1180A/ES260 blends were also studied in the present work (Figure 5a,b). It can be seen from Figure 5 and Table 3 that ES260 has the highest hardness and tensile strength while exhibiting a relatively low elongation at break. ESC85A and ET1180A show smaller tensile strength but larger elongation at break. For the blends, mechanical properties always changed between neat TPUs; the overall trend was that with increasing ES260 content, the tensile strength improves, while the elongation at break gradually decreases. It is noteworthy that for the ESC85A/ES260 blend system (Figure 5a), the yield strength of ESC85A/ES260 = 7:3 increased by almost 143% relative to neat ESC85A. By contrast, the yield strength of ET1180A/ES260 = 7:3 (Figure 5b) increased by only about 44% relative to neat ET1180A. The elongation at break for ESC85A/ES260 = 5:5 (Figure 5a) was also higher than that of ET1180A/ES260 = 5:5. Since ESC85A and ES260 both have ester-based soft segments, PBS and PBT (listed in Table 1), and similar hard segment content (Table 1), the molecular chains of the two components present better compatibility. Thus, in the blend system, the aggregation structures of the two kinds of chains rearranges at the molecular level. The hard segment domain of the ESC85A/ES260 blend comprises both ESC85A and ES260 hard segments, forming new phase separation structures with larger phase region distances (Figure 3a). The hard segments with better mechanical properties in ES260 can be better dispersed in the blend substrate, resulting in a significant increase in yield strength. However, the ET1180A contains lower hard segment and ether based soft segment content. The different chemical structure leads to a relatively lower compatibility with ester based ES260. The hard domains of the ET1180A/ES260 blend, though mutually integrated to some extent, still partly maintain their respective micro domains, illustrated in Figure 3b. Thus, although the yield strength of ET1180A/ES260 = 7:3 is improved, the increase is not obvious. For the elongation at break, the better compatibility and complete rearrangement of the phase separation structure contributes to a better interface of the ESC85A/ES260 blends (5:5), and ultimately represents a larger elongation at break than that of ET1180A/ES260. The latter decreases significantly with the ratio of 5:5.

Table 3 also shows the hardness of TPU blends. ES260 has the largest Shore D hardness (52.7) of the three brands. For the blend systems, the hardness of blends increases as the ES260 content increases. That indicates the hardness of designed material can be easily control by TPU blends.

### 3.5. Atomic Force Microscopy (AFM) Phase Images of TPU and Their Blends

**Figure 6:**
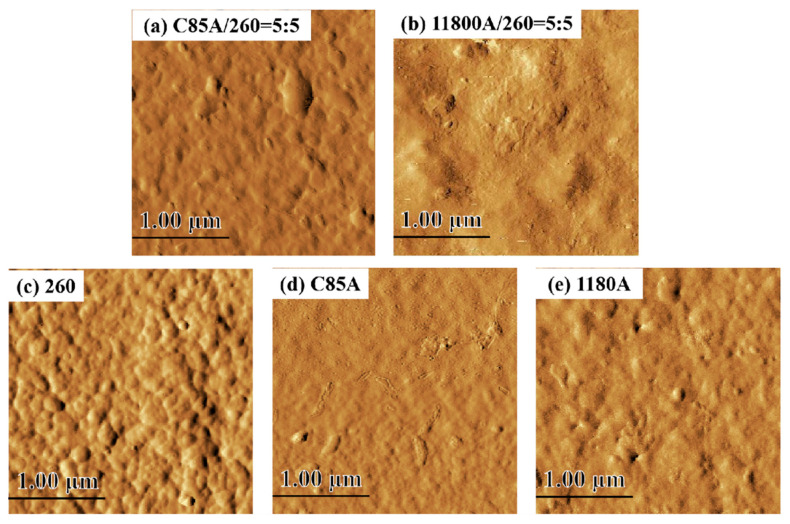
AFM phase images comparing the phase area size change of TPU blends at 50% ratio (**a**) ESC85A/ES260 = 5:5, (**b**) ET1180A/ES260 = 5:5 and neat TPU (**c**) ES260, (**d**) ESC85A, (**e**) ET1180A. The smaller swollen particles in (a) indicate that they have a larger phase domain size. All scanning areas were 2.5 × 2.5 μm^2^ with 29.469 nm roughness (Rq).

Figure 6 shows the AFM phase images of neat TPU and 50% TPU blend system with a scanning area of 2.5 μm × 2.5 μm. The sample’s surface was etched in tetrahydrofuran (THF) solvent for 30 min to remove the soft segment phase, leaving the hard segment as the dominant one, shown in Figure 6 as a bulge. The spacing of the grains in the bulges indicates the distance between the hard segments [36]. The bulging particles in the ESC85A/ES260 blend system are small, as shown in Figure 6a. The results show that compared to the ET1180A/ES260 blend system (Figure 6b), the former has a larger phase zone size, consistent with the data in SAXS. Similarly, the neat TPU ES260 (Figure 6c) has more raised particles and a higher content of hard segments. This shows that the phase area size of ES260 is larger than that of ESC85A (Figure 6d) and ET1180A (Figure 6e). The changes in these phase regions validates the conclusions drawn in SAXS.

## 4. Conclusions

In this work, TDI-polyester based TPU was blended with MDI-polyether based TPU and MDI-polyester based TPU, respectively. The difference in the microphase separation structure of the two TPU blend systems led to the difference of compatibility at the molecular level, which affected the thermal and mechanical properties.

DSC study show that in the two blending systems, the hard segment of the ES260 component could be mutually integrated with ESC85A and ET1180A, respectively, resulting in a significant reduction of the melting temperature of the ES260 component in the blend system.

SAXS results presented a formation of new microphase separation structures of ESC85A/ES260 blends. Since ESC85A and ES260 both have ester based soft segments, PBS and PBT, and close hard segment content, the molecular chains of the two components present better compatibility. Thus, in the blend system, the aggregation structure of the two kinds of chains rearranges at the molecular level. However, the ET1180A has a lower hard segment and ether based soft segment content. The different chemical structure leads to a relatively lower compatibility with ester based ES260. The hard domains of the ET1180A/ES260 blend, though mutually integrated to some extent, still partly maintain their respective micro domains. The AFM results confirmed the change in the size of the phase area detected by SAXS. The better compatibility makes the hard domains of ESC85A/ES260 blends mutually integrate to form new phase separation structures with larger phase region distances. 

With the change of the microphase separation structures, the blend system also showed differences in mechanical properties. The yield strength of the 70/30 ESC85A/ES260 blend increased by almost 143%, but the elongation at break only reduced by 12%. By contrast, the yield strength of 70/30 ET1180A/ES260 increased by only about 44%. The elongation at break of 50/50 ET1180A/ES260 decreased as the blend partly maintained its respective micro domains, forming weak interfaces.

This work discussed the difference of phase separation structures of TPU blend systems, providing guidance for the design of TPU properties by adjusting microphase structure.

## Figures and Tables

**Table 1:** Hard segment content (HSC), Shore hardness of materials, molecular weight, Mw/Mn, melt flow rate and compositions of three neat TPU.

Sample	Diisocyanate	Diols	HSC (wt.%)	Shore Hardness	Mw (×10^5^ g·mol^−1^)	Mw/Mn	Melt Flow Rate (g·10 min^−1^) *
ES260	TDI	PBT	33	60D	1.57	3.12	29.8
ESC85A	MDI	PBS	33	87A	2.57	3.32	33.3
ET1180A	MDI	PTHF	25	80A	1.83	2.17	22.4

* The melt flow rate was tested at 220 °C.

**Table 2:** Compositions of TPU blends and moulding temperatures.

ESC85A/ES260 Blends (wt.%)	ET1180A/ES260 Blends (wt.%)	Moulding Temperature (°C)
100/0	100/0	210
70/30	70/30	210
50/50	50/50	215
30/70	30/70	220
0/100	0/100	220

**Table 3:** Mechanical property data of neat TPU and TPU blends.

Scheme	Yield Strength (MPa)	Elongation at Break (%)	Shore D Hardness
ES C85A	3.5 ± 0.7	644.7 ± 30.1	29.5 ± 0.2
ET 1180A	3.6 ± 0.3	638.7 ± 27.5	27.2 ± 0.5
ES 260	18.2 ± 0.1	395.6 ± 25.6	52.7 ± 0.9
ES C85A/ES 260 = 7:3	8.5 ± 0.3	562.1 ± 30.5	38.5 ± 0.7
ES C85A/ES 260 = 5:5	11.8 ± 0.2	520.4 ± 28.4	47.0 ± 0.2
ES C85A/ES 260 = 3:7	11.9 ± 0.4	502.9 ± 29.3	50.0 ± 0.4
ET 1180A/ES 260 = 7:3	5.4 ± 0.6	525.6 ± 29.7	31.5 ± 0.3
ET 1180A/ES 260 = 5:5	7.3 ± 0.3	423.8 ± 26.4	38.8 ± 0.4
ET 1180A/ES 260 = 3:7	14.3 ± 0.5	469.2 ± 27.3	46.3 ± 0.7

## Data Availability

All the data are available within this manuscript.
